# Context Is Everything: Harmonization of Critical Food Microbiology Descriptors and Metadata for Improved Food Safety and Surveillance

**DOI:** 10.3389/fmicb.2017.01068

**Published:** 2017-06-26

**Authors:** Emma Griffiths, Damion Dooley, Morag Graham, Gary Van Domselaar, Fiona S. L. Brinkman, William W. L. Hsiao

**Affiliations:** ^1^Department of Molecular Biology and Biochemistry, Simon Fraser University, VancouverBC, Canada; ^2^Department of Pathology and Laboratory Medicine, University of British Columbia, VancouverBC, Canada; ^3^National Microbiology Laboratory, Public Health Agency of Canada, WinnipegMB, Canada; ^4^Department of Medical Microbiology and Infectious Diseases, Max Rady College of Medicine, University of Manitoba, WinnipegMB, Canada; ^5^British Columbia Centre for Disease Control Public Health Laboratory, VancouverBC, Canada

**Keywords:** genomic epidemiology, foodborne pathogen surveillance, outbreak investigations, ontology, contextual metadata

## Abstract

Globalization of food networks increases opportunities for the spread of foodborne pathogens beyond borders and jurisdictions. High resolution whole-genome sequencing (WGS) subtyping of pathogens promises to vastly improve our ability to track and control foodborne disease, but to do so it must be combined with epidemiological, clinical, laboratory and other health care data (called “contextual data”) to be meaningfully interpreted for regulatory and health interventions, outbreak investigation, and risk assessment. However, current multi-jurisdictional pathogen surveillance and investigation efforts are complicated by time-consuming data re-entry, curation and integration of contextual information owing to a lack of interoperable standards and inconsistent reporting. A solution to these challenges is the use of ‘ontologies’ - hierarchies of well-defined and standardized vocabularies interconnected by logical relationships. Terms are specified by universal IDs enabling integration into highly regulated areas and multi-sector sharing (e.g., food and water microbiology with the veterinary sector). Institution-specific terms can be mapped to a given standard at different levels of granularity, maximizing comparability of contextual information according to jurisdictional policies. Fit-for-purpose ontologies provide contextual information with the auditability required for food safety laboratory accreditation. Our research efforts include the development of a Genomic Epidemiology Ontology (GenEpiO), and Food Ontology (FoodOn) that harmonize important laboratory, clinical and epidemiological data fields, as well as existing food resources. These efforts are supported by a global consortium of researchers and stakeholders worldwide. Since foodborne diseases do not respect international borders, uptake of such vocabularies will be crucial for multi-jurisdictional interpretation of WGS results and data sharing.

## Introduction: the Importance of Metadata and Contextual Information in Foodborne Safety and Surveillance

Foodborne pathogens impact global health and can cost economies millions of dollars in lost productivity (Flynn, 2014; Minor et al., 2015; World Health Organization, 2015). “Integrated surveillance” combines data from different stages of the farm-to-fork food continuum to provide multi-sector information for infectious disease surveillance, and represents the most comprehensive strategy to improve food safety (Zaidi et al., 2008; Ammon and Makela, 2010; Danan et al., 2011). Central to public health microbiology, food safety, and disease surveillance activities, is the comparison of genetic relatedness between isolates from human, food, and environmental samples. Whole genome sequencing (WGS) provides the highest resolution evidence for inferring phylogenetic relationships among foodborne pathogens (Ashton et al., 2016; Kanagarajah et al., 2017; Waldram et al., 2017). However, genomic sequences can only be consistently interpreted for food safety and surveillance when the data are linked to standardized, fit-for-purpose contextual information suitable for use by data analysts, data consumers, and stakeholders (Lambert et al., 2017).

*Contextual information* in genomic epidemiology investigations includes critical knowledge about sequencing pipelines and sequence quality, sources of exposure and risk, clinical phenotypes, susceptible populations, geographical distribution and more. Reliable capture of parameters pertaining to sample provenance (specimen types and sources), sample processing (DNA extraction and sequencing library construction), quality control (sequence quality and contamination detection), data analysis (bioinformatic pipelines) are critical for reproducibility, comparability, and calibration of genomic results (Kircher et al., 2011; Paszkiewicz et al., 2014; Lynch et al., 2016). In addition to sequencing and bioinformatics parameters, laboratory test results characterizing antimicrobial resistance and virulence phenotypes often reveal important pathogen determinants that help to inform source and risk (World Health Organization, 2008; Clark et al., 2016; Glasset et al., 2016; Sharma et al., 2016; Day et al., 2017; Kanengoni et al., 2017; Tagini et al., 2017). Clinical information about the host, and epidemiological information about possible exposures (high-risk food types), are all useful to establish at-risk populations and hypothesize about likely sources of contamination (World Health Organization, 2008). This information is also used to establish the geographic distribution of pathogenic strains, as well as among populations, which is critical for determining transmission patterns (Moura et al., 2016; Njamkepo et al., 2016). Rich contextual information increases the utility of genomics data used for food safety surveillance, outbreak investigations, source attribution and risk assessments. Risk analysis in particular requires precise data on pathogen hazards in food to be systematically linked to epidemiological data, in order to make assessments, implement interventions and monitor outcomes (Lammerding and Fazil, 2000; Hoornstra et al., 2001; Food and Agriculture Organization of the United Nations [FAO], 2005).

Unfortunately, resource-demands for the collection of such information, inconsistencies in descriptors, as well as other political and technical barriers have proven to complicate data sharing and integration between agencies. Wide adoption of contextual information best practices, as well as storage and sharing practices, would enable rapid, on-demand comparison of sequences from different sources and agencies, enhancing pathogen detection, inter-agency communication and responses. Here, we describe these various challenges and explain how informatics innovations such as ontologies can provide much needed solutions to streamline data interpretation and exchange for improved food safety and public health.

## Barriers To Integration and Sharing of Whole Genome Sequence Data and Contextual Information

Despite a growing global commitment to the use and sharing of public health microbiology data, implementation at local, regional, national, and international levels has proven challenging with both political and technological barriers (van Panhuis et al., 2014). Fundamental structural barriers embedded in public health governance systems arise as the result of lack of trust (Pisani and AbouZahr, 2010; Fidler and Gostin, 2011; van Panhuis et al., 2014). Perceptions of risk to patient privacy and intellectual property, as well as the fear of misinterpretation and potential misuse of data are some of the biggest challenges to the sharing of sequence data and the exchange of contextual information (van Panhuis et al., 2014). Risk aversion practices prompt health agencies to implement blanket policies restricting data sharing, which result in incomplete metadata attached to sequences in public data repositories (van Panhuis et al., 2014).

**FIGURE 1:**
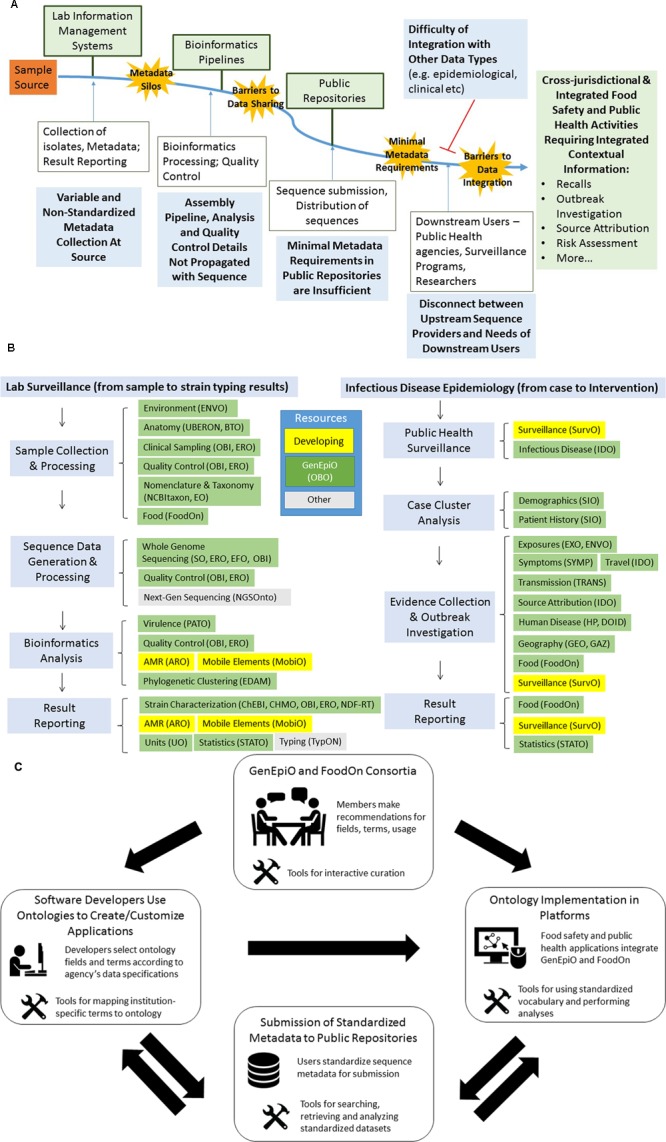
The political and technological barriers to propagating contextual information with genomics sequences. Fit-for-purpose contextual information must be integrated for optimal food safety and public health activities such as surveillance, recalls, outbreak investigations, source attribution, risk assessments and so on. Lab Information Management Systems (LIMS) are often the point-of-entry of samples into the genomics data flow pipeline. Variability in contextual information collection occurs as LIMS often do not conform to the recommendations of minimal information checklists. Collected information is recorded as free text, agency-specific shorthand and often documented in paper format, all of which contribute to the formation of metadata silos. Bioinformatics processing, phylogeny construction, inference and interpretation are often carried out by different analysts, and software parameters are rarely propagated with genomic data. Restrictive governance and data sharing policies protecting patient privacy and intellectual property of data can reduce the amount of metadata categories and content submitted to public repositories. Repositories, such as those of the International Nucleotide Sequence Database Collaboration (NCBI, EMBL-EBI, DDBJ) have recognized the need for harmonized metadata, and have committed to adopting a minimal metadata standard (Minimal Data for Matching (Global Microbial Identifier (2013)). While MDM field requirements are a progressive step, metadata details are entered as non-standardized free text, which require time-consuming curation to integrate with other types of data. These technical and political barriers hinder the potential use of genomic sequences in complex food safety activities and contribute to delayed results and uncertainty in analyses. **(B)** GenEpiO imports terms from compatible OBO Foundry ontologies, enabling data harmonization and integration across data types. Fit-for-purpose contextual information is essential to fully exploit the potential of WGS data, and to carry out regulatory and public health activities such as product traceback and outbreak investigations. Standardized vocabulary offered by ontologies facilitates auditability, attribution, usability and clarity of contextual information, and the reuse of terms and universal IDs better enable integration of information across sectors and domains of information. Furthermore, ontologies can empower the programmatic characterization of genomics clusters (e.g., food products and exposures, demographics, symptoms, geography, AMR, virulence) using different data types generated by different health and regulatory bodies. To standardize information regarding microbial typing and lab surveillance, as well as infectious disease epidemiology, GenEpiO imports vocabulary and logic from over 25 different existing ontologies. Subsets of fields and terms derived from these ontologies describe sample collection and processing, sequence data generation and processing, bioinformatics analysis, public health surveillance, case cluster analysis, outbreak investigation and result reporting. Ontologies listed in green represent OBO Foundry ontologies, which can be found at http://www.obofoundry.org/. Ontologies listed in yellow, are currently under development by the authors and associated consortia (ARO, MobiO, SurvO). Resources listed in grey represent other useful non-OBO ontologies (http://bioportal.bioontology.org/ontologies). **(C)** The mobilization of GenEpiO and FoodOn ontologies. Mobilization of GenEpiO and FoodOn ontologies can only be achieved by consensus and wide adoption. As such, domain experts of the GenEpiO and FoodOn international consortia will make curation and term recommendations to ensure proper usage and sufficiency of vocabulary. User-friendly tools, with training instructions, are being created to better enable users to interact with the ontologies. Furthermore, tools currently in development for enabling software developers to select subsets of fit-for-purpose fields and terms will enable the construction of applications and platforms designed to handle and analyze harmonized contextual information (e.g., IRIDA). Ontology logic can be used to flag fields of data for security and privacy issues, thereby reducing risk. Standardized datasets can be submitted to public repositories, which can be more extensively queried. The requirement for ontology implementation by accreditation bodies will better enable the calibration of datasets between labs, and facilitate regulation.

Technological barriers for electronic data interchange exacerbate issues of political distrust (van Panhuis et al., 2014). Contextual data are mostly expressed as free text or agency-specific terminology. While reports and guidelines exist in an effort to suggest minimum contextual information that should be attached to genomic sequences, these fields are rarely incorporated into Lab Information Management Systems (LIMS) and epidemiology surveillance forms (Field et al., 2014; Grad and Lipsitch, 2014; Aziz et al., 2015; McMahon and Denaxas, 2016; Lambert et al., 2017). Through user interviews and needs assessments, we and others have found that information is then “siloed” in different hard drives, agencies, in restrictive data formats (paper or antiquated electronic formats), and is often collected for short-term purposes (van Panhuis et al., 2014). Owing to such inconsistency, recoding of the data is often needed for data sharing across institutions participating in multi-jurisdictional surveillance, impacting response time. By relying on retrospective retrieval from different sources (as opposed to real-time collection), the quality and quantity of contextual information become eroded over time. Flow of contextual information from source to end user, as well as barriers to collection and sharing are illustrated in **Figure 1**.

## Existing Resources For Metadata Standardization and Food Safety: From Checklists To Ontologies

One of the biggest challenges to the standardization of metadata capture for food safety is the large number of incompatible food classifications used worldwide. These food classifications range from lists of food types, descriptors of food production environments, codes of practice, guidelines, and other recommendations relating to foods, food production, and food safety. While these resources are certainly useful, they have been developed for specific uses, and fundamental differences in their architecture limit interoperability. A selection of such food dictionaries can be found in **Table 1**. For example, analyses of foodborne outbreak data for source attribution requires the categorization of reported food vehicle. Variation in the way aetiological agents and foods are defined and categorized, even within a single country or jurisdiction, has been shown to impede direct comparison of food attribution across countries within similar time periods (Greig and Ravel, 2009). While up-to-date food safety best practices prescribe data collection systems to be sufficiently precise in order to minimize uncertainty, in reality, inconsistencies in descriptors pertaining to the host, pathogen, environment, and the underlying attributes of potentially contaminated foods, all contribute to uncertainty in data analyses and delay in public health action (Greig and Ravel, 2009).

**Table 1:** A selection of ontology and Minimum Information (MI) checklists for the standardization of genomics metadata and epidemiological, clinical, and laboratory contextual information.

Resource	Description	URL
Codex Alimentarius	• Internationally recognized standards, codes of practice, guidelines • Recommendations relating to foods, food production, and food safety • Commissioned by the United Nations Food and Agriculture Organization	http://www.fao.org/fao-who-codexalimentarius/codex-home/en/
LanguaL	• Created by US FDA’s Centre for Food Safety and Applied Nutrition • 14 main facets, or hierarchies of descriptive terms (35,000 foods) • Available in many languages.	http://www.langual.org/
Food Ex2	• Created by the European Food Safety Authority (EFSA) • Food classification designed to facilitate food exposure assessment	https://www.efsa.europa.eu/en/data/data-standardisation
USDA National Nutrient Database for Standard Reference	• Food dictionary containing over 9000 foods • Each item lists nutrient values and weights per portion	https://ndb.nal.usda.gov/ndb/foods
Compendium of Analytical Methods	• Created by Health Canada • Food list containing several hundred items organized by food category • Designed to foster compliance of the food industry with standards and guidelines relative to microbiological and extraneous material in foods	http://www.hc-sc.gc.ca/fn-an/res-rech/analy-meth/microbio/volume1-eng.php
Food Commodity Classification Scheme	• Created by the US Center for Disease Control • Designed for source attribution studies	http://www.ncbi.nlm.nih.gov/pubmed/19968563
The Agriculture Ontology (AgrO)	• The ontology of agronomic practices, agronomic techniques, and agronomic variables used in agronomic experiments	http://www.obofoundry.org/ontology/agro.html
Antimicrobial Resistance Ontology (ARO)	• Ontology of antibiotics, resistance genes, and associated phenotypes	https://card.mcmaster.ca/
Basic Formal Ontology (BFO)	• Upper level ontology designed to support information retrieval, analysis and integration in scientific, and other domains	http://www.obofoundry.org/ontology/bfo.html
BRENDA Tissue Ontology (BTO)	• Structured controlled vocabulary for the source of an enzyme comprising tissues, cell lines, cell types, and cell cultures	http://www.obofoundry.org/ontology/bto.html
Chemical Entities of Biological Interest Ontology (ChEBI)	• Structured classification of molecular entities of biological interest focusing on ‘small’ chemical compounds	http://www.obofoundry.org/ontology/chebi.html
Cell Ontology (CL)	• Structured controlled vocabulary for cell types in animals	http://www.obofoundry.org/ontology/cl.html
Human Disease Ontology (DOID)	• Ontology for describing the classification of human diseases organized by etiology	http://www.obofoundry.org/ontology/doid.html
EMBRACE Data and Methods Ontology (EDAM)	• Ontology of common bioinformatics operations, topics, types of data including identifiers, and formats	http://www.ontobee.org/ontology/EDAM
Environment Ontology (ENVO)	• Contained descriptors of a range of food products and food production environments • Limited in scope, based on user suggestions	http://www.obofoundry.org/ontology/envo.html
Epidemiology (EPO)	• Ontology designed to support the semantic annotation of epidemiology resources	http://www.obofoundry.org/ontology/epo.html
Exposure (EXO)	• Vocabularies for describing exposure data to inform understanding of environmental health	http://www.obofoundry.org/ontology/exo.html
Foundational Model of Anatomy (FMA)	• Ontology representing phenotypic structures of the human body	http://www.obofoundry.org/ontology/fma.html
FooDB Ontology (FoodO)	• Designed to represent the FooDB database describing food items and chemical composition (additives, ingredients, etc)	http://aber-owl.net/ontology/FOODO
Food Ontology (FoodOn)	• Farm-to-Fork descriptors of food entities and food production environments from point of production through processing, distribution and consumption • Created by the FoodOn Consortium	http://www.obofoundry.org/ontology/foodon.html http://foodontology.github.io/foodon/
Genomic Epidemiology Ontology (GenEpiO)	• Controlled vocabulary for infectious disease surveillance and outbreak investigations implementing whole genome sequencing • Ongoing development via the International GenEpiO Consortium	http://www.genepio.org http://www.obofoundry.org/ontology/genepio.html
Infectious Disease Ontology (IDO)	• Ontology describing entities relevant to both biomedical and clinical aspects of most infectious diseases	https://bioportal.bioontology.org/ontologies/IDO
Next-Generation Sequencing Ontology (NGSOnto)	• Structured vocabulary to capture the workflow of all the processes involved in a Next Generation Sequencing project	https://bioportal.bioontology.org/ontologies/NGSONTO
Ontology for Biomedical Investigations (OBI)	• Ontology for the description of life-science and clinical investigations	http://www.obofoundry.org/ontology/obi.html
Phenotypic Quality Ontology (PATO)	• Ontology of biomedical phenotypic qualities (properties, attributes or characteristics)	http://www.obofoundry.org/ontology/pato.html
Relation Ontology (RO)	• Biology-specific relations to connect entities and classes • Intended for standardization across OBO Foundry Library of ontologies	http://www.obofoundry.org/ontology/ro.html
The Sustainable Development Goals Interface Ontology (SDGIO)	• The Sustainable Development Goals Interface Ontology of United Nation Environmental Program	https://github.com/SDG-InterfaceOntology/sdgio
Sequence Ontology (SO)	• Structured controlled vocabulary for sequence annotation, for the exchange of annotation data and for the description of sequence objects in databases	http://www.obofoundry.org/ontology/so.html
Systematized Nomenclature of Medicine (SNOMED)	• Represents clinical phrases captured by the clinician • Created by The International Health Terminology Standards Development Organisation (IHTSDO)	http://www.ihtsdo.org/snomed-ct
Clinical Signs and Symptoms Ontology (SYMP)	• Ontology to provide robust means to disambiguate, capture and document clinical signs, and symptoms	http://www.obofoundry.org/ontology/symp.html
Pathogen Transmission Ontology (TRANS)	• Ontology for describing transmission methods of human disease pathogens, from one host, reservoir, or source to another host	http://www.obofoundry.org/ontology/trans.html
Microbial Typing Ontology (TypOn)	• Structured vocabulary to describe microbial typing methods for the identification of bacterial isolates and their classification	https://bioportal.bioontology.org/ontologies/TYPON
Multi-Species Anatomy Ontology (UBERON)	• Integrated cross-species anatomy ontology covering animals and bridging multiple species-specific ontologies	http://www.obofoundry.org/ontology/uberon.html
MIxS	• A minimal metadata standard checklist developed by the Genomic Standards Consortium (GSC) for reporting information about any (x) nucleotide sequence	Yilmaz et al., 2011
Project and Sample Application Standard	• Created by the National Institute of Allergy and Infectious Disease Genome Sequencing Center and Bioinformatics Resource Center (GSCID/BRC) • Specifically addresses metadata types that should be attached to human pathogen genomic sequences	Dugan et al., 2014
Minimum Information about a Phylogenetic Analysis (MIAPA)	• Community-wide effort to develop minimal reporting standards for phylogenetic analyses	Leebens-Mack et al., 2006
STROME-ID guidelines	• “Strengthening the reporting of molecular epidemiology for infectious diseases” • Standards for reporting molecular epidemiology results including measures of genetic diversity, laboratory methods, sample collection, etc	Field et al., 2014
The Global Alliance for Genomics and Health (GA4GH)	• Aim to create a common, harmonized framework to enable secure sharing of genomic and clinical data	http://genomicsandhealth.org/
The Global Microbial Identifier (GMI)	• Platform for storing whole genome sequencing (WGS) data of microorganisms to detect outbreaks and emerging pathogens	http://www.globalmicrobialidentifier.org/
The United Nations Environment Programme (UNEP)	• Leading global environmental authority • Promotes the coherent implementation of actions for sustainable development (Sustainable Development Goals)	http://web.unep.org/
United Nations Environment Live	• Interactive platform for environmental assessments and peer review of the SDGIO	https://uneplive.unep.org/sdgs

In designing an approach to capture standardized metadata, it is critical to define what information about a sample is most informative for its intended use. This process is best achieved via engagement of a variety of end users - in this case food regulators, epidemiologists, lab analysts, bioinformaticians, at local, regional, national and international levels. Minimum Information (MI) checklists represent the sum of all essential data fields recommended by community experts and users, with controlled vocabularies used as ‘allowed values’ (Field and Sansone, 2006). A well-known genomic metadata standard is the MIxS checklist, a minimal metadata standard checklist developed by the Genomic Standards Consortium (GSC) for reporting information about any nucleotide sequence (Yilmaz et al., 2011). Similarly, the National Institute of Allergy and Infectious Diseases Genome Sequencing Center and Bioinformatics Resource Center (GSCID/BRC) Project and Sample Application Standard specifically addresses metadata types that should be attached to human pathogen genomic sequences (Dugan et al., 2014). Additionally, the Minimum Information about a Phylogenetic Analysis (MIAPA) represents a community-wide effort to develop minimal reporting standards for phylogenetic analyses (Leebens-Mack et al., 2006). These checklists contain a wide variety of descriptive fields; however, they currently lack standardized values to enter in the fields.

A more comprehensive mechanism for making metadata searchable and actionable, is through the use of ’ontologies’ (Bodenreider and Stevens, 2006; Brinkman et al., 2010). Ontologies are hierarchies of well-defined and standardized vocabulary interconnected by logical relationships (Bodenreider and Stevens, 2006). These logical interconnections provide a layer of intelligence to query engines, making ontologies much more powerful than simple flat lists of terms. Terms and their definitions, are specified by universal IDs (Universal Resource Identifiers), which associate descriptors with particular usages and disambiguate meaning (Bodenreider and Stevens, 2006). Ontologies also incorporate synonyms of terms in the definitions and identifiers (IDs) e.g., biscuits (United Kingdom) and cookies (North America), enabling institutions to use their preferred terminology while simultaneously mapping terms to an ontology standard. The hierarchical structure enables comparison of entities at different levels of granularity (e.g., leafy greens and spinach), which represents an important feature for evolving food safety investigations in which the hypothesized food vehicle is a moving target. Mapping to an ontology-based standard and reuse of universal IDs makes software implementing the ontology framework interoperable, enabling faster and more efficient data exchange (Arp et al., 2015). The reuse of terms and their IDs enables integration of different data types across domains (epidemiology, food, disease, agriculture, antimicrobial resistance, etc) and between agencies (Ferreira et al., 2013). Computer and human readable (in different natural languages), ontology hierarchies allow stakeholders to share data according to the level of granularity permitted by jurisdictional policies, and fields of information with legal or privacy issues can be flagged using ontology relations to increase security. Furthermore, fit-for-purpose ontologies provide contextual information with the auditability required for food safety and public health laboratory accreditation (Evans, 2015). Principles of good practice in ontology development have been put into practice within the framework of the Open Biomedical Ontologies consortium through its OBO Foundry initiative, which emphasizes collaborative development, interoperability and usability (Smith et al., 2007). Descriptors of genomic epidemiological processes have already been captured in a number of existing ontologies. Some examples include the Sequence Ontology (SO) (Eilbeck et al., 2005), the EDAM Bioinformatics Ontology (EDAM) (Ison et al., 2013), and DOID (Schriml et al., 2012), which describe sequences, genome assembly, and human disease. The Exposure, Epidemiology, Environment, Symptoms, and Transmission Ontologies (EXO, EPO, ENVO, SYMP, TRANS) describe types of exposures, facets of epidemiology, natural and built environments, clinical signs and symptoms, and modes of transmission (Mattingly et al., 2012; Pesquita et al., 2014; Buttigieg et al., 2016). Ontologies and other resources useful for genomic epidemiology are listed in **Table 1**.

Currently, no resource(s) integrate all the necessary components of a genomic epidemiology investigation. As such, our research efforts have focused on the development of a Genomic Epidemiology Ontology (GenEpiO), based on public health stakeholder interviews and the harmonization of important laboratory, clinical and epidemiological data fields, in collaboration with a consortium of researchers and end users. We are also actively developing, in collaboration with members of the international GenEpiO consortium, a Farm-to-Fork food ontology (FoodOn) aiming to harmonize existing food resources and describe food entities from point(s) of production/collection, through processing, distribution and consumption.

## GenEpiO and FoodOn: New Developments in Food Safety Semantics

The Genomic Epidemiology Ontology (GenEpiO) is an ontology resource being developed according to the principles of the OBO Foundry, led by a partnership of Canadian scientists representing academic, provincial and federal public health interests. The objective of GenEpiO is to enable integration and propagation of all necessary contextual information required to interpret microbial pathogen genomics data, from the point-of-sample-intake, through sequencing, to end use (e.g., during a foodborne outbreak investigation). The GenEpiO hierarchy was constructed based on the Basic Formal Ontology (BFO) and Relation Ontology (RO) of the OBO Foundry, which delineate how *things* should be organized into higher level *classes*, and how *things* and *classes* should relate to one another (Smith et al., 2005; Arp et al., 2015). This architecture improves compatibility with other OBO biomedical ontologies, enriching vocabulary and data linkages, and facilitating the reuse of terminology and the integration of information across health and food safety domains (agriculture, veterinary care, environment, food production). The considerable consensus achieved by the OBO Foundry has paved the way for harmonization of complex content in a way that is unavailable with other disparate ontologies. GenEpiO terms are mapped to community standards and over 25 existing ontologies to ensure the accuracy of meaning and to facilitate interoperability (**Figure 1B**). GenEpiO also includes data models comprising disease/agency/reporting or analytical system/surveillance network-specific fields, which can be used to represent genomic epidemiology workflows, processes, disease progression and decision-making. GenEpiO currently contains over 2000 key fields and terms to harmonize sample metadata, lab analytics, wet lab and bioinformatics processes, quality control, clinical information as well as exposures and epidemiological data. As such, we anticipate that GenEpiO will better enable the calibration and validation of genomics for clinical and regulatory use. Controlled vocabulary and relationship logic are encoded in the Web Ontology Language, OWL. OWL files are publicly available, and can be implemented in different software applications (**Table 1**). The GenEpiO ontology is currently being implemented within the Integrated Rapid Infectious Disease Analysis (IRIDA) platform^1^, an open source, secure web-based, end-to-end platform for infectious disease genomic epidemiology, spearheaded in Canada. Within IRIDA, GenEpiO is being used to generate NCBI BioSample-compliant submission-ready genome metadata files, and to create different Line List visualization tools for epidemiological investigations. The next phase of development will involve the complete integration of GenEpiO to enhance the platform’s analytical power.

FoodOn encompasses materials in natural ecosystems, as well as human-centric food items, food production environments and handling of food (Griffiths et al., 2016). We aim to develop semantics for food safety, food security, the agricultural and animal husbandry practices linked to food production, culinary, nutritional and chemical ingredients and processes. As such, FoodOn architecture is similarly based on BFO and RO schema, as well as the facet-based LanguaL (*Langua aLimentaria*, or language of food) classification system of the US Food and Drug Administration (US FDA) (Ireland and Møller, 2010). Facets include Food Products, which can be linked to Food Sources, Cooking and Preservation Methods, Consumer Groups, Cultural Origins, Taxonomy and more. Thousands of individual food products have already been indexed according to the LanguaL system, and are publicly available in a separate FoodOn import file (**Table 1**). The scope of FoodOn is ambitious and will require input and long-term development by multiple domain experts. Further details regarding GenEpiO and FoodOn design and content will be discussed elsewhere (manuscripts in preparation).

In order to ensure utility, accuracy and usability, user engagement is a top priority for GenEpiO and FoodOn development. Feedback from engagement efforts has indicated that user-friendly tools for curation of terms, implementation, and mapping between interfaces and agencies, would serve to mobilize these technologies. To that effect, we are currently developing software applications for ontology mapping and curation. Additionally, both ontologies can be searched using various widely used portals such as the EBI Ontology Look-up Service, Ontobee, and NCBO BioPortal (**Table 1**). As harmonization of the both GenEpiO and FoodOn ontologies can only be achieved by consensus and wide adoption, involving open source and open access initiatives, we have catalyzed the formation of international consortia to build partnerships and solicit contributions from domain experts. The GenEpiO consortium membership comprises over 70 participants from 15 countries, with leadership, technical and editorial working groups. The interaction of the consortia, tools, applications, ontologies, users and repositories will be important for soliciting term contributions, as well as integrating regional- and sector-specific vocabulary, and evolving strategies for international uptake (**Figure 1C**).

## Broader Context of Food Genomics Metadata and Ontologies

Several frameworks for integrating genomics and other data currently exist for tackling the real-world problems of emerging diseases, environmental degradation, world hunger, and sustainability. Each of these global partnerships seeks to streamline the flow of genomics knowledge and its application for solving global challenges. The Global Alliance for Genomics and Health (GA4GH) and The Global Microbial Identifier (GMI) work to establish common frameworks and transdisciplinary networks to better monitor and control emerging public health threats (Knoppers, 2014; Wielinga et al., 2017). The Environmental Working Group of the United Nations (UNEP) have developed Sustainable Development Goals addressing climate change, renewable energy, food, health and water provision requiring the coordinated global monitoring (United Nations, 2016). Each of these efforts involves highly negotiated language representing different disciplines and policies, which can be harmonized into a coherent system through the use of ontologies. GA4GH and UNEP currently implement OBO Foundry ontologies that have been integrated into GenEpiO (e.g., ENVO, UBERON, ChEBI). GenEpiO integrates the Minimal Data for Matching standards for matching pathogen isolates prescribed by the GMI consortium (Global Microbial Identifier, 2013), and GenEpiO and FoodOn standards are being considered for an upcoming ISO (International Organization for Standards) guideline on the use of WGS for Food Safety. The standardized food and food environment descriptors being developed in FoodOn can fill a critical gap in community standards required to integrate food related data in each of these efforts. Global initiatives and associated ontologies can be found in **Table 1**. Public health and genomics descriptors found in GenEpiO, combined with existing compatible ontologies for describing different environments (ENVO), agriculture (AgrO), and sustainable development (SDGIO), will greatly enable the integration of knowledge required to accomplish global health, equity and sustainability goals (**Table 1**).

## Conclusion

Platforms implementing ontologies such as GenEpiO and FoodOn will be the work-engines ensuring the integration and reusability of genomics data from the collection of samples, through consumption by various end users. With the international nature of food distribution and food safety concerns, the most effective semantic resources must be open source, interoperable and collaboratively developed in order to best represent the needs of the international community. Global networks navigating the political challenges inherent in such community efforts will be crucial for the success of genomics as the new currency of food and waterborne pathogen typing. While no “one-size-fits-all” data dictionary for genomic epidemiology currently exists, harmonization of different vocabularies can be achieved through the use of ontologies and the flexibility they provide. With growing support of community-based development efforts, this foundational work can facilitate intra- and international data exchange, resulting in improved food safety and health outcomes globally, as well as promoting innovation and discovery.

## Author Contributions

EG wrote the manuscript. EG and DD developed software, concepts and resources. MG and GVD contributed input, use cases and testing material for resource development. WH and FB conceived the project and supervised this work. DD, MG, GVD, FB, and WH provided feedback on the manuscript.

## Conflict of Interest Statement

The authors declare that the research was conducted in the absence of any commercial or financial relationships that could be construed as a potential conflict of interest.

## Footnotes

1 www.irida.ca
